# Efficacy of sequential TACE on primary hepatocellular carcinoma with microvascular invasion after radical resection: a systematic review and meta-analysis

**DOI:** 10.1186/s12957-023-03160-0

**Published:** 2023-09-05

**Authors:** Anwei Mo, Biquan Lin, Denglin Chen

**Affiliations:** 1 Department of Medical Oncology, Hainan General Hospital (Hainan Affiliated Hospital of Hainan Medical University), Haikou, Hainan China; 2 Intervention Clinic, Hainan General Hospital (Hainan Affiliated Hospital of Hainan Medical University), No. 19 Xiuhua Road, Haikou, Hainan 570000 China

**Keywords:** Hepatocellular carcinoma, Radical resection, Transcatheter arterial chemoembolization, Microvascular invasion

## Abstract

**Objectives:**

The purpose of this study is to examine the impact of sequential transcatheter arterial chemoembolization (TACE) on the prognosis of patients with hepatocellular carcinoma (HCC) and microvascular invasion (MVI) following radical resection.

**Methods:**

Five databases were searched for studies on the efficacy of TACE after radical hepatectomy resection (HR) for treating HCC with MVI. Depending on the heterogeneity between included studies, the relative risk (RR) and 95% confidence interval (CI) were computed using a random or fixed effect model.

**Results:**

Thirteen articles were included in this study. There were 1378 cases in the HR-TACE group (cases undergoing TACE after HR) and 1636 cases in the HR group (cases only undergoing HR). The recurrence-free survival (RFS) at 1 year, 2 years, 3 years, and 5 years after radical HCC resection was statistically significantly greater in the HR-TACE group than in the HR group. The HR-TACE group exhibited statistically significant advantages at 1-year, 2-year, 3-year, and 5-year overall survival (OS) after radical HCC resection when compared with the HR group.

**Conclusion:**

Postoperative sequential TACE treatment can improve the RFS and OS rates at 1 year, 2 years, 3 years, and 5 years following radical HR in patients with HCC and MVI. These findings will guide clinicians in selecting appropriate cases for adjuvant TACE treatment during clinical diagnosis and treatment to maximize patient benefit.

**Trial registration:**

PROSPERO CRD42023449238.

**Supplementary Information:**

The online version contains supplementary material available at 10.1186/s12957-023-03160-0.

## Introduction

Primary liver cancer is a highly lethal malignant tumor [1, 2]. Hepatocellular carcinoma (HCC) is the most prevalent pathological form of primary liver cancer, accounting for approximately 90% of cases in adults [1–3]. The clinical characteristics of HCC are its insidious onset and high mortality. Surgical intervention remains the preferred method for treating HCC [2–4]. Comprehensive treatment by multiple measures is also an essential option for HCC patients [4].

TACE exploits the biological fact that the hepatic artery is the primary source of blood supply to HCC tumor tissue. Through the puncture catheter, chemotherapy drugs and vascular embolization agents are injected into the blood supply vessels of the tumor to form a high concentration of chemotherapy drugs in the tumor and surrounding tissues. Besides, it could build an environment where the blood supply of HCC tumor tissue is inadequate to treat HCC tumors. TACE is an essential treatment option for patients with HCC who are unable or unwilling to undergo surgical resection or have recurrence after surgical treatment [5].

With the enhancement of surgical instruments and the further development of medical research, the overall prognosis and survival of patients with HCC after radical resection have been significantly improved. Patients with microvascular invasion (MVI)-positive HCC have a higher risk of relapse [6, 7]. According to the relevant literature, MVI positivity is one of the independent risk factors for postoperative recurrence in patients with HCC [8, 9]. Currently, surgeons rely primarily on postoperative pathology to confirm the diagnosis of MVI, which causes a delay in the formulation of preoperative, individualized treatment plans and surgical procedures for HCC patients.

It is debatable whether postoperative prophylactic adjuvant TACE should be conducted on patients with MVI. Zhong et al. suggested that postoperative adjuvant TACE treatment, which involves the inactivation of residual micro-metastatic active lesions, has significant benefits in reducing the postoperative recurrence rate in patients with MVI positivity [10]. In contrast, according to Zhang et al., adjuvant TACE cannot benefit patients undergoing radical resection for HCC. Even the chemotherapy medications in TACE treatment will decrease individual immunity and promote early tumor recurrence [11]. The follow-up treatment options for pathology-confirmed MVI after radical hepatectomy (HR) have received less attention in previous research.

Based on this background, this study searched the literature comparing sequential TACE after radical HR and radical HR alone in HCC patients with MVI positivity. We explored the effect of sequential TACE on the survival of patients with HCC who were MVI-positive after radical resection. This meta-analysis aimed to provide evidence-based medical evidence for the clinical application of TACE in the adjuvant treatment of HCC patients with MVI positivity after radical resection.

## Methods

This systematic review and meta-analysis have been registered at PROSPERO (registration number: CRD42023449238) (https://www.crd.york.ac.uk/PROSPERO/).

### Search strategy

PubMed, The Cochrane Library, Web of Science, CNKI, and Wanfang Data were searched for the efficacy of TACE in the treatment of HCC with MVI after radical HR. “Hepatocellular Carcinoma,” “Liver, Neoplasms,” “Microvascular Invasion, MVI,” “Transarterial Chemoembolization,” and “transcatheter arterial chemoembolization, TACE” were the keywords in this search. The references of systematic review and meta-analyses were screened again. All the retrieved articles were subjected to independent, double-blind selection. The retrieval time was from the establishment of each database to January 2023.

### Literature inclusion and exclusion

Two researchers independently and double-blindly reviewed the title, abstract, and full text of the literature to include the eligible literature in accordance with the inclusion and exclusion criteria. After the screening, the full texts of all included literature were meticulously perused for assessing the quality of the study. Low-quality literature was subsequently excluded. If the opinions of the researchers were inconsistent, the third experts were consulted to undergo a rigorous discussion and quality evaluation in determining the quality of the argued study. Inclusion criteria: MVI was verified by pathology in the subjects after radical resection of liver carcinoma (only MVI; no large vascular invasion or other metastasis). The patient’s condition was satisfactory, and no other interventions were performed before surgery. There were randomized controlled studies, and observational studies, including cohort studies, and case–control studies among the categories of literature studies. During the observation period, the only intervention measures were TACE intervention or no intervention. No other adjuvant treatment measures, such as sorafenib adjuvant therapy, were administered concurrently. Before the occurrence of the outcome indicators, the patients enrolled in the study did not receive any other treatment besides the investigated intervention. In addition, the clinically fundamental characteristics did not differ significantly between the two groups in the study. This meta-analysis selected 1-year recurrence-free survival (RFS), 2-year RFS, 3-year RFS, and 5-year RFS, 1-year overall survival (OS), 2-year OS, 3-year OS, and 5-year OS as outcome indicators. The exclusion criteria for this meta-analysis were the following: there was imaging evidence of metastasis or other malignant tumors, as well as other organic diseases that might impact an individual’s survival. Patients had imaging or pathological evidence of extensive tumor thrombus invasion of blood vessels. Patients received sorafenib targeted therapy before or after radical resection of HCC and sequential TACE after radical resection. The study’s samples were less than thirty. The follow-up was inadequate, and the attrition rate was greater than 10%. The studies were lack of original data, such as abstracts, meta-analyses, expert opinions, and case reports, and were lack of a control group. Poor quality and repeated publications were excluded from this meta-analysis.

### Quality assessment of included studies

The quality of the included literature was evaluated independently by two researchers. If the evaluations of the researchers were inconsistent, a third party should be involved in a robust discussion. The Newcastle–Ottawa Scale (NOS) was used to assess the quality of the cohort studies included in the analysis [12]. To evaluate the literature, the NOS scale was divided into three sections (object selection, comparability, and outcome evaluation) that were further subdivided into eight items. The total tally was nine points, with 7–9 points representing excellent quality, 4–6 points representing medium quality, and 1–3 points representing low quality. After eliminating low-quality studies, a meta-analysis was conducted.

### Data extraction and aggregation

Two researchers independently extracted from the included research the basic information of the literature, clinical characteristics of the two groups, and outcome indicators. Two researchers cross-checked the extracted data. If the extracted data were inconsistent, re-extraction of the data was required and a third party was consulted to discuss the reasons. All information was extracted from the tables and graphs provided by the literature. The extracted data included general information about the literature (article title, first author, publication date, country or region, research type, etc.), data necessary for assessing the quality of the literature, general information about the subjects (sample size, age, gender, tumor size, number of tumors, TACE regimen, and times, etc.), and various observation indicators (RFS at 1 year, 2 years, 3 years, and 5 years after surgery, OS at 1 year, 2 years, 3 years, and 5 years after surgery).

### Statistical analysis

For statistical analysis, STATA 15.1 software was used [13, 14]. In this study, the *I*^*2*^ value was used to quantitatively evaluate whether or not there was heterogeneity and heterogeneity between the included literature: *I*^*2*^ < 25% was judged to be no heterogeneity, *I*^*2*^ < 50% was judged to be mild heterogeneity, *I*^*2*^ < 75% was judged to be moderate heterogeneity, and *I*^*2*^ ≥ 75% was judged to be high heterogeneity. If there was moderate heterogeneity (*I*^*2*^ < 50%, *P* > 0.1), the fixed effect model was utilized to derive the overall statistics. If *I*^*2*^ ≥ 50%, *P* ≤ 0.1, it was assumed that there was evident heterogeneity among the included literature, and the random effect model was used to calculate the combined statistics. The relative risk (RR) and its 95% confidence interval (CI) were used to illustrate the outcomes of the meta-analysis. If *P* > 0.05, the Egger linear regression test indicated no publication bias; if *P* ≤ 0.05, however, there was publication bias [15]. The trim and fill test of Duval and Tweedie was used to determine the sensitivity of the results. Suppose the combined effect size was spliced before and after the test, and the combined effect size changed significantly. In that case, the research results were unreliable and that additional analysis of the spliced research was required [16]. Unless *P* < 0.001, we would provide an exact value for *P*.

## Results

### Literature screening process, basic characteristics, and quality evaluation of included studies

**Fig. 1:**
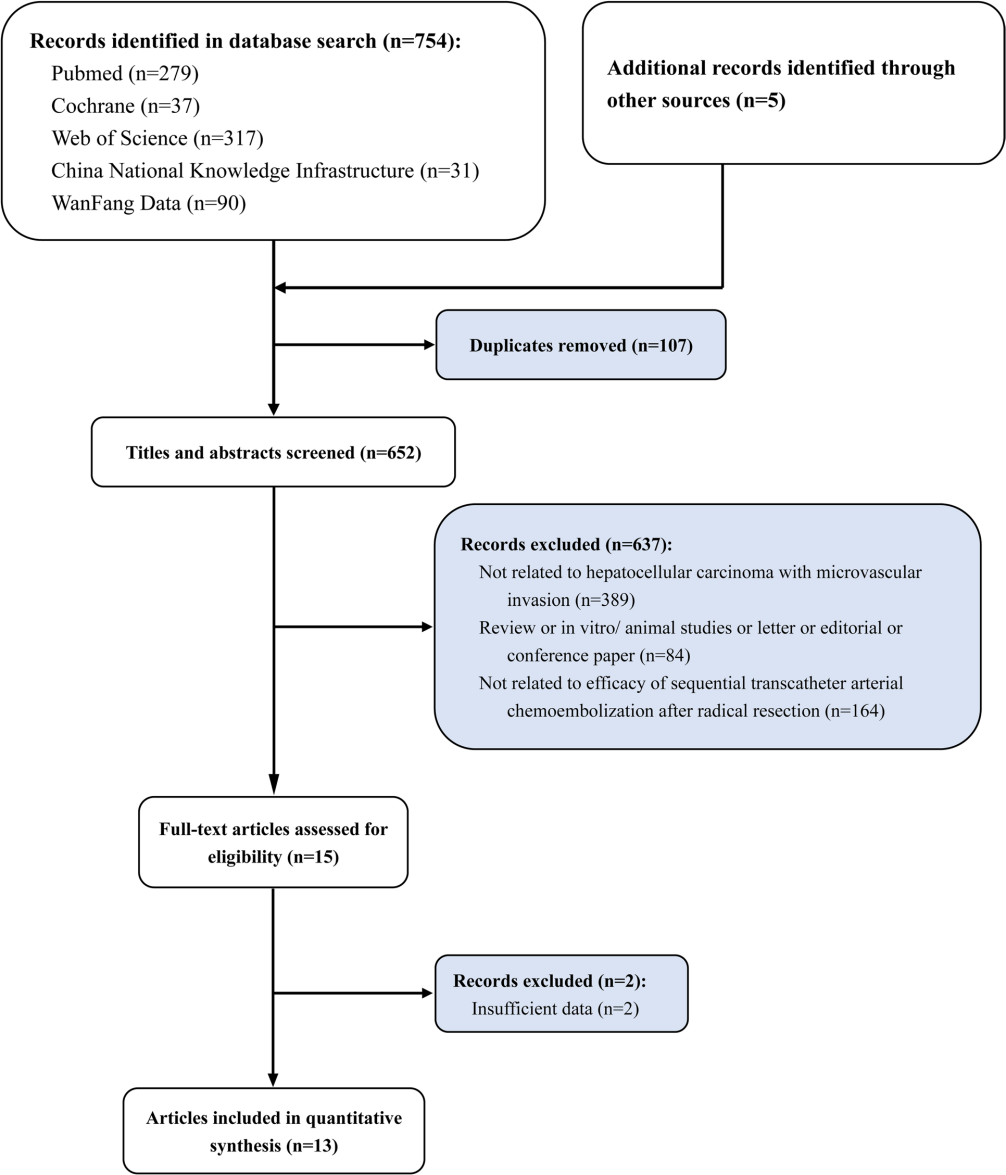
Study selection flowchart

From the database’s inception until January 2023, we retrieved 759 relevant publications from numerous databases. By perusing the article’s title and abstract, 671 irrelevant articles were eliminated. According to the study’s predetermined inclusion and exclusion criteria, 73 of the 88 studies were excluded due to a lack of original data, inconsistent interventions, non-controlled trial studies, insufficiently related outcome indicators, and repetitive literature. The quality of the remaining 15 articles that met the requirements was assessed, and two low-quality articles were eliminated. In the end, thirteen articles were included for meta-analysis in the study (Fig. 1). All of them were retrospective cohort studies. The literature’s characteristics were detailed in Table 1. 3014 patients were included in the study, 1378 of whom were assigned to the HR-TACE group and 1636 to the HR group [17–29]. The retrospective cohort studies with NOS scores of 6 or higher were identified as high-quality literature (Table 1 and Supplemental Table 1).

**Table 1:** Baseline characteristics of included studies for meta-analysis

First author, year	Groups	No. of cases	Sex (male/female)	Diameter of tumor (< 5 cm/ ≥ 5 cm)	Complete tumor capsule (yes/no)	Differentiated degree (1–2/3–4)	NOS score
Wei W, 2019 [17]	HR-TACE	116	106/10	0/116	NA	NA	6
	HR	118	106/12	0/118	NA	NA	
Tian BY, 2018 [18]	HR-TACE	95	84/11	44/51	55/40	53/42	8
	HR	205	177/28	89/116	123/82	107/98	
Liu JH, 2016 [19]	HR-TACE	137	125/12	5.00 ± 1.30	87/50	75/62	7
	HR	159	134/25	8.00 ± 3.80	140/19	102/57	
Liu ZY, 2017 [20]	HR-TACE	62	90/17	6.30 ± 2.27	56/6	24/38	6
	HR	45		6.50 ± 2.41	40/5	15/30	
Shen PC, 2020 [21]	HR-TACE	52	37/15	27/25	24/28	20/32	7
	HR	49	36/13	30/19	21/28	23/26	
Qi YP, 2019 [22]	HR-TACE	91	78/13	27/25	59/32	NA	7
	HR	109	93/16	30/19	61/48	NA	
Li KW, 2012 [23]	HR-TACE	35	39/2	14/21	25/10	24/11	7
	HR	41	32/3	15/26	28/13	30/11	
Ye JZ, 2017 [24]	HR-TACE	86	75/11	41/45	51/35	49/37	9
	HR	174	150/24	76/98	105/69	91/83	
Wang L, 2020 [25]	HR-TACE	199	176/23	85/114	134/65	184/15	8
	HR	199	173/26	83/116	137/62	184/15	
Wang H, 2018 [26]	HR-TACE	44	42/2	3.84 ± 1.27	31/13	1/43	9
	HR	84	76/8	3.83 ± 1.09	50/34	6/78	
Sun JJ, 2016 [27]	HR-TACE	137	120/17	6.51 ± 0.27	11/126	13/124	9
	HR	185	167/18	6.99 ± 0.29	17/168	10/175	
Wang YY, 2019 [28]	HR-TACE	57	47/10	6 (2–14)	NA	41/16	7
	HR	57	51/6	6 (2–18)	NA	41/16	
Liu S, 2019 [29]	HR-TACE	222	193/29	111/111	99/123	147/75	8
	HR	222	193/29	101/121	105/117	131/91	

*Abbreviation*: *NOS* Newcastle Ottawa Scale, *HR-TACE* cases undergoing transcatheter arterial chemoembolization after hepatectomy resection, *HR* cases only undergoing hepatectomy resection

### Meta-analysis results of outcome indicators

#### Postoperative 1-year, 2-year, 3-year, and 5-year RFS

**Fig. 2:**
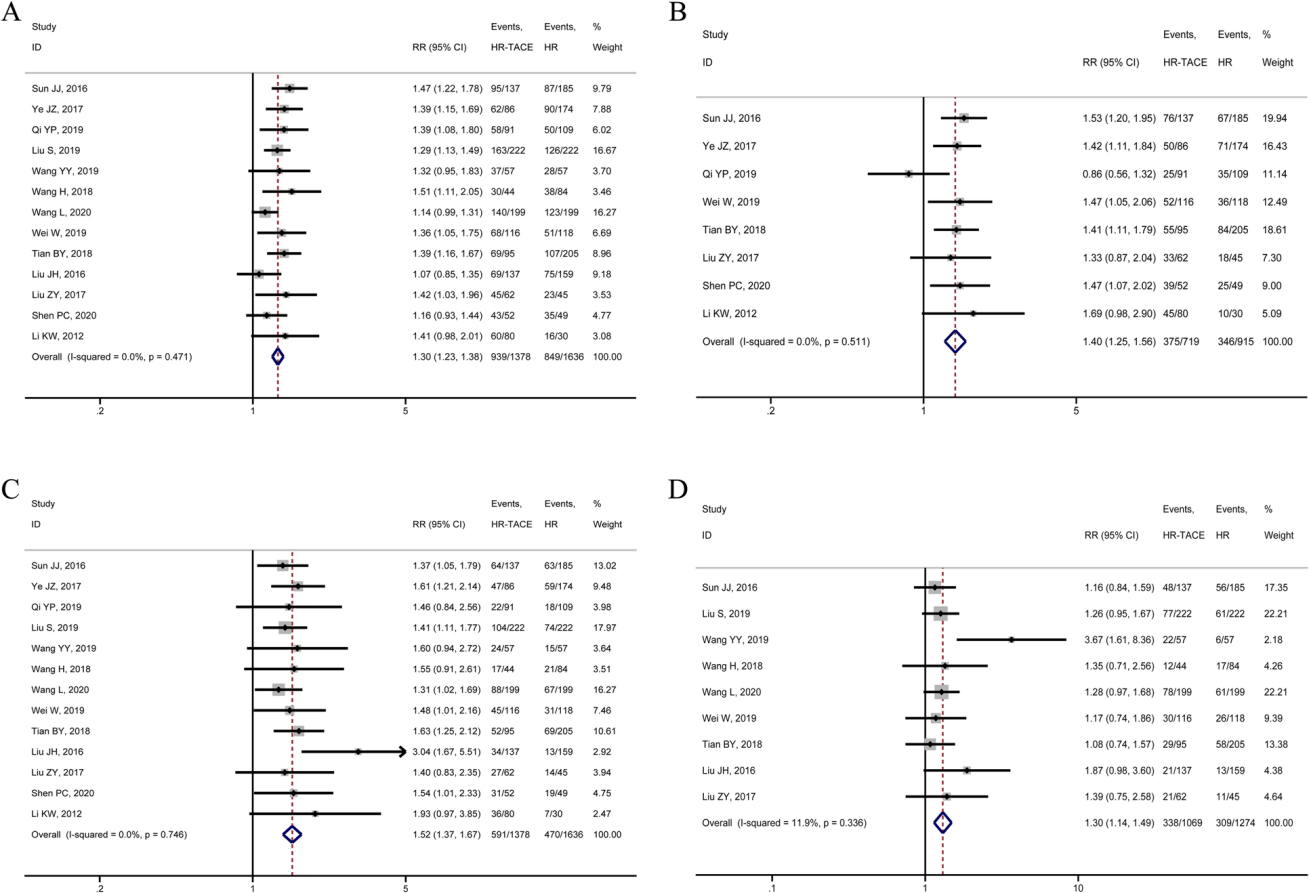
Forest plots of meta-analysis results of outcome indicators: **A** 1-year RFS; **B** 2-year RFS; **C** 3-year RFS; **B** 5-year RFS; RFS recurrence-free survival

Thirteen articles investigated the 1-year and 3-year RFS rate after radical HCC resection. A total of 1378 cases and 1636 cases were in the HR-TACE group and HR group, respectively. The results of the heterogeneity test revealed no statistically significant heterogeneity between included studies (RFS at 1 year after operation: *P* = 0.471, *I*^*2*^ = 0.0%; RFS at 3 years after operation: *P* = 0.746, *I*^*2*^ = 0%; Fig. 2A and C). The results of the fixed-effects model showed that the RFS of the HR-TACE group was better than that of the HR group at 1 year and 3 years after radical resection of HCC, and the difference was statistically significant (RFS at 1 year after operation: RR = 1.30, 95%CI 1.23–1.38; RFS at 3 years after surgery: RR = 1.52, 95%CI 1.37–1.67; Fig. 2A and C). Eight articles involving 1634 patients reported the 2-year RFS rate after radical resection of hepatocellular. The HR-TACE group had 719 cases, while the HR group had 915 cases. The heterogeneity test results indicated no statistically significant heterogeneity (RFS 2 years after surgery: *P* = 0.511, *I*^*2*^ = 0.0%; Fig. 2B). At 2 years after radical resection of HCC, the meta-analysis results of the fixed effect model suggested that the RFS of the HR-TACE group was superior to that of the HR group (RFS: RR = 1.40, 95%CI 1.25–1.56; Fig. 2B). Nine articles including 1069 cases in the HR-TACE group and 1274 cases in the HR group reported 5-year RFS. No statistically significant heterogeneity was observed between studies (RFS 5 years after operation: *P* = 0.336, *I*^*2*^ = 11.9%; Fig. 2D). The HR-TACE group presented higher 5-year RFS than that of the HR group, and the difference was statistically significant (RFS at 5 years after surgery: RR = 1.30, 95%CI 1.14–1.49; Fig. 2D).

#### Postoperative 1-year, 2-year, 3-year, and 5-year OS

**Fig. 3:**
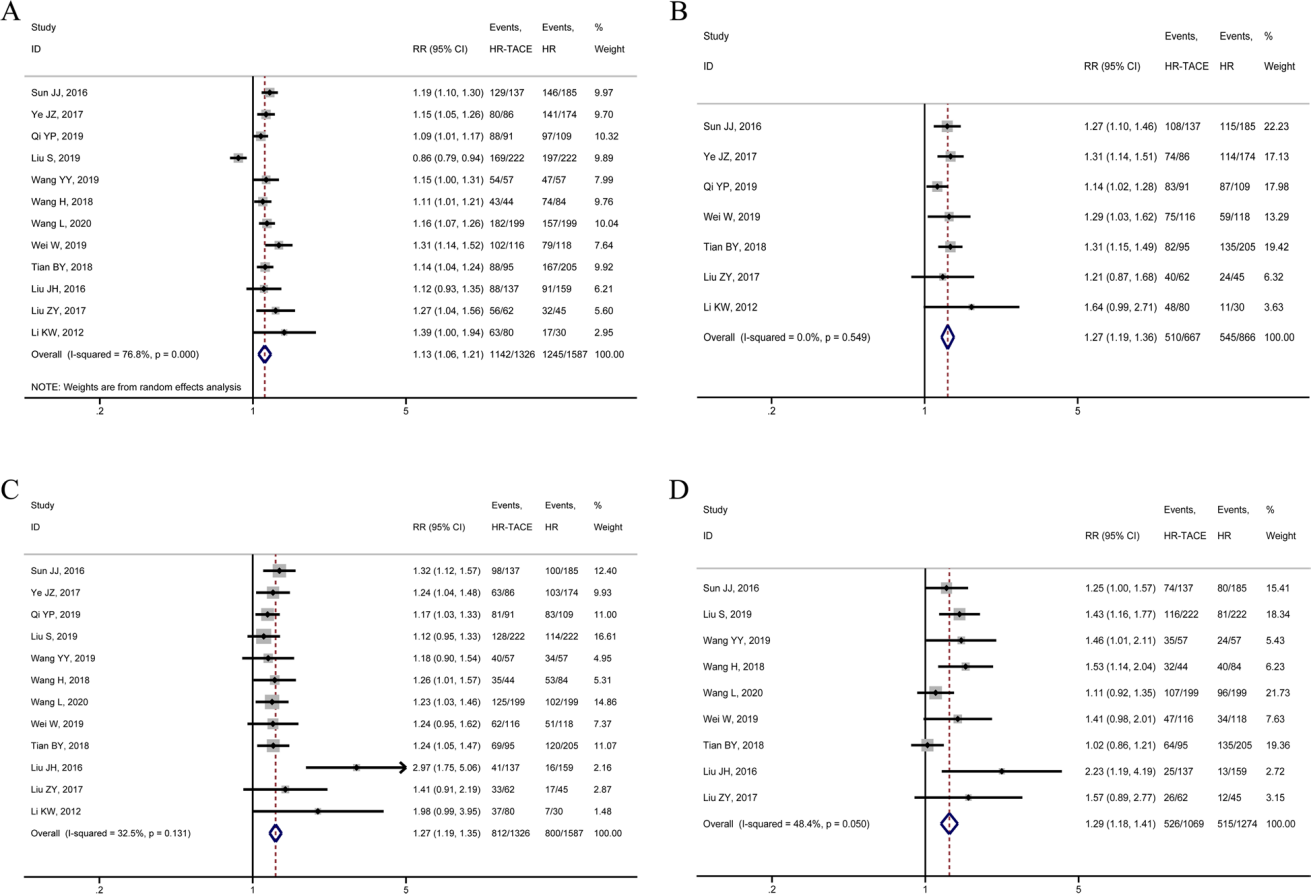
Forest plots of meta-analysis results of outcome indicators: **A** 1-year OS; **B** 2-year OS; **C** 3-year OS; **B** 5-year OS; OS overall survival

Thirteen articles reported the OS after radical resection of HCC in this meta-analysis. There were 12 articles, 7 articles, 12 articles, and 9 articles describing the 1-year, 2-year, 3-year, and 5-year OS after radical HCC resection, respectively. The 1-year and 3-year OS rates after radical resection of HCC were calculated for 2913 patients, 1326 of whom were in the HR-TACE group and 1587 in the HR group. In consideration of statistically significant heterogeneity in studies reported 1-year OS after radical resection of HCC, random-effects model meta-analysis results showed that the HR-TACE group was superior to the HR group with statistically significant difference (RR = 1.13, 95%CI 1.06–1.21; Fig. 3A). With no substantial intergroup heterogeneity was observed in 3-year OS, 3-year OS after radical resection of HCC in the fixed effect model showed that HR-TACE gained better OS than HR alone (RR = 1.27, 95%CI 1.19–1.35; Fig. 3C). Similarly, with an appropriate statistical method, the 2-year and 5-year OS rates of patients in the HR-TACE group after radical HCC resection were higher than those in the HR alone group (2-year OS after surgery: RR = 1.27, 95%CI 1.19–1.36; 5-year OS after surgery: RR = 1.29, 95%CI 1.18–1.41; Fig. 3B and D).

### Subgroup analysis

**Fig. 4:**
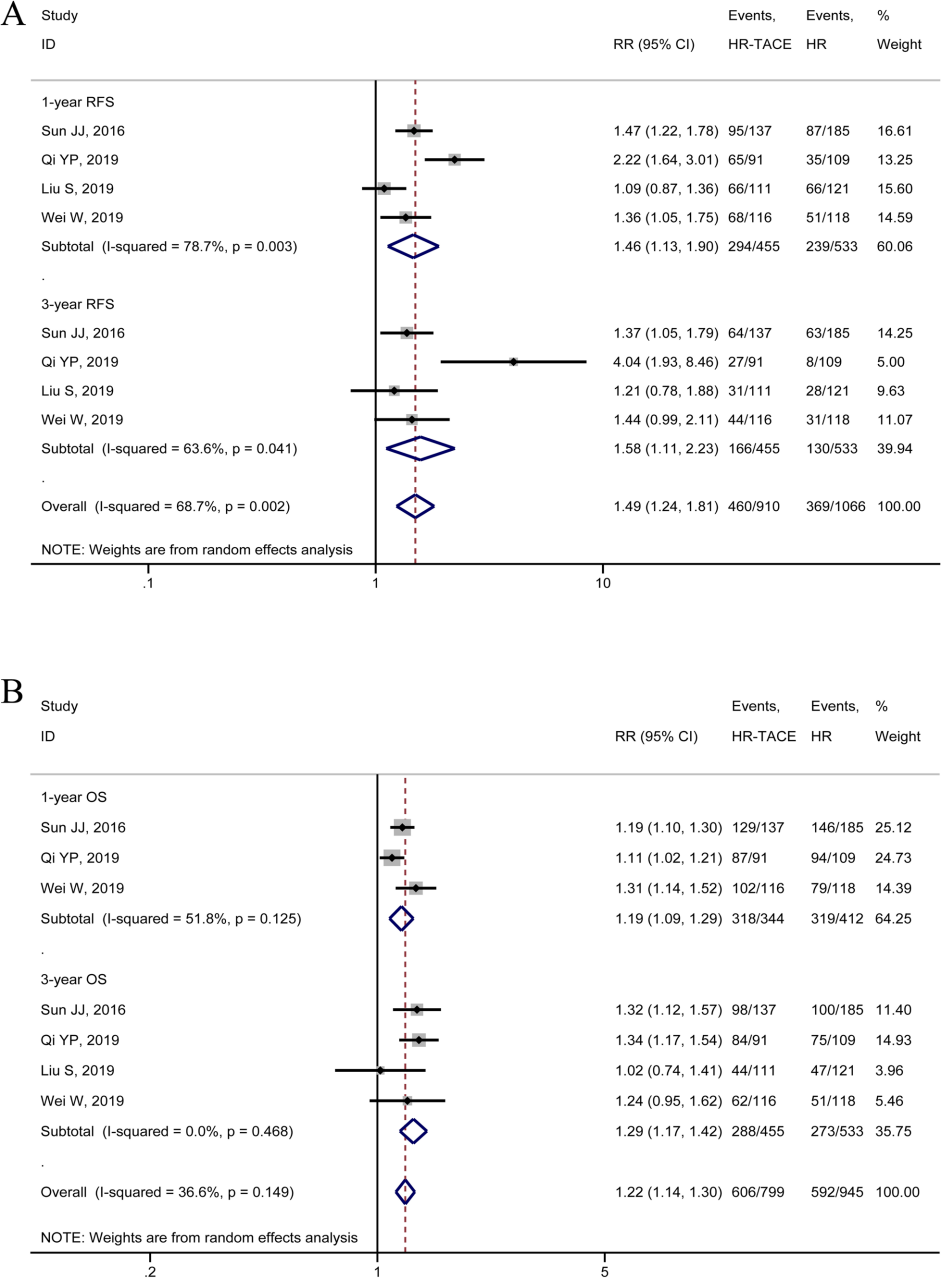
Forest plots of meta-analysis results of subgroup analysis: **A** 1-year RFS and 3-year RFS in patients with HCC which tumor diameter equal or greater than 5 cm; **B** 1-year OS and 3-year OS in patients with HCC which tumor diameter equal or greater than 5 cm; RFS, recurrence-free survival; HCC, hepatocellular carcinoma; OS, overall survival

Four of the 13 included studies reported the 1-year and 3-year RFS and OS after radical resection of HCC patients which tumor diameter equal to or greater than 5 cm and MVI. A subgroup analysis was conducted on RFS and OS at 1 year and 3 years after radical resection of HCC. The results showed that the RFS of the HR-TACE group was better than that of the HR group at 1 year and 3 years after the operation, and the difference was statistically significant (RFS at 1 year after operation: RR = 1.46, 95%CI 1.13–1.90; 3-year RFS after operation: RR = 1.58, 95%CI 1.11–2.23; Fig. 4A). The 1-year and 3-year OS of the HR-TACE group was statistically superior to that of the HR group (1-year OS after operation: RR = 1.19, 95%CI 1.09–1.29; 3–year OS: RR = 1.29, 95%CI 1.17–1.42, Fig. 4B). The findings of the meta-analysis indicated that sequential TACE treatment after radical resection of HCC was preferable to radical resection of HCC in terms of RFS and OS at 1 year and 3 years after surgery for HCC patients with MVI positivity and tumor diameter ≥ 5 cm.

### Publication bias and sensitivity analysis

This study used Egger’s test to detect publication bias in the meta-analysis of sequential TACE treatment for HCC patients with MVI positivity after radical resection. Significant publication bias was observed in the postoperative 3-year and 5-year OS. The limited sample size in some of the included studies influence the meta-analysis results (Table 2). In addition, the effect sizes of the outcome indicators discussed in this meta-analysis did not vary significantly before and after Duval and Tweedie’s trim and fill sensitivity test (Table 2). Both of them confirmed the efficacy of sequential TACE therapy in patients with MVI-positive HCC after radical resection.

**Table 2:** Evaluation of publication bias and sensitivity analysis

Index	Egger’s regression		Duval and Tweedie’s trim and fill		
	Intercept	*p*	Original effect size	Studies trimmed	Adjusted effect size
1-year RFS	1.465	0.232	1.31 (1.24, 1.39)	4	1.27 (1.19, 1.35)
2-year RFS	− 1.127	0.494	1.42 (1.29, 1.54)	1	1.40 (1.28, 1.53)
3-year RFS	2.348	0.074	1.58 (1.41, 1.75)	0	1.58 (1.41, 1.75)
5-year RFS	3.688	0.061	1.47 (1.16, 1.78)	0	1.47 (1.16, 1.78)
1-year OS	2.486	0.196	1.14 (1.07, 1.21)	5	1.08 (1.01, 1.14)
2-year OS	1.319	0.265	1.26 (1.19, 1.33)	2	1.24 (1.17, 1.31)
3-year OS	3.728	0.012	1.33 (1.19, 1.47)	3	1.22 (1.05, 1.39)
5-year OS	3.875	0.007	1.36 (1.19, 1.54)	4	1.21 (1.04, 1.39)

*Abbreviation*: *RFS* Recurrence-free survival, *OS* Overall survival

## Discussion

Clinically, in some cases of early HCC recurrence after surgery, no vascular invasion or metastases were detected during the preoperative examination, while MVI was confirmed in pathology after surgery. According to relevant studies, MVI is one of the independent risk factors for the early recurrence of HCC following radical resection [8, 9]. There is still no consensus regarding the necessity of sequential adjuvant TACE therapy for patients with HCC who have undergone radical resection and had MVI confirmed by pathology following radical resection. The RFS and OS rates are indispensable outcomes for determining patients’ long-term prognosis and quality of life. They serve as essential indicators for assessing the efficacy of therapy. At 1 year, 2 years, 3 years, and 5 years after radical resection of HCC, the RFS of the HR-TACE group was superior to that of the HR group, and the difference was statistically significant. Similarly, the HR-TACE group exhibited a statistically significant advantage over the HR group in the 1-year, 2-year, 3-year, and 5-year OS following radical resection of HCC. The analysis results revealed that radical resection followed by TACE therapy not only exhibited an anti-tumor recurrence effect but also provided a survival advantage for HCC patients with MVI positivity.

We hypothesize that sequential TACE therapy can prevent tumor recurrence and enhance the OS time of HCC patients with MVI positivity after radical resection for the following reasons: First, hepatic angiography is useful for the early detection of microsatellite lesions during TACE therapy. Nevertheless, preoperative imaging examination and the intraoperative naked eye cannot find them. And concurrent chemoembolization can increase postoperative survival. Second, following radical resection of HCC, intrahepatic occult-free HCC cells may rapidly proliferate and relapse in the residual liver parenchyma while stimulating multiple growth factors. TACE therapy embolizes the blood supply vessels of recurrent tumors, cuts off the nutrients necessary for tumor growth, and inhibits the proliferation of tumor cells. Third, the anticancer effects of chemotherapeutic medications on the proliferation and apoptosis of HCC cell clones are prominent. Fourth, the accelerated proliferation of residual cancer cells increases their sensitivity to chemotherapeutic medications, which may be one of the reasons why the HR-TACE group has a higher recurrence-free survival rate than the HR group. Fifth, TACE treatment mainly increases the concentration of local chemotherapy drugs in the liver, and its postoperative complications and adverse events are significantly lower than systemic intravenous chemotherapy, which has a relatively small impact on patient’s quality of life and improves survival. Lastly, the commonly used chemotherapy drugs for adjuvant TACE, such as lobaplatin, are metabolized by the liver to an almost negligible degree. The drug interaction is minor, so it will not substantially increase the hepatic parenchyma burden. It will not enormously impair liver function in patients with HCC who have undergone surgery and has a minimal impact on patients’ long-term survival.

Many scholars believe that tumor size is not only an obvious indicator of disease severity but also a crucial factor influencing patient survival and prognosis. The greater the diameter of the tumor, the greater the likelihood of early postoperative recurrence. It has been documented that when the diameter of HCC is ≥ 5 cm, the probability of MVI positivity is between 70 and 90% [8, 30]. During surgical resection of multiple or large nodular tumors, micro-metastases may have spread beyond the surgical margin. Pathology-confirmed MVI increases the likelihood of detecting microsatellite nodules and postoperative recurrence. It will result in a dismal prognosis following surgery. This study conducted a subgroup analysis on the cases with tumor diameters ≥ 5 cm. The results demonstrated that sequential TACE treatment after radical HCC resection was more effective at 1 and 3 years after surgery for patients with HCC with tumor diameter ≥ 5 cm and MVI. Both RFS and OS were preferable to HCC radical resection alone.

This research also has some limitations (1) All of the included studies were retrospective cohort studies. Therefore, the quality of the evidence must be enhanced. (2) There was no exhaustive and detailed subgroup analysis of the included studies for distinct population characteristics. Future large-sample, multicenter, long-term follow-ups with rigorous designs are required to confirm the curative efficacy of TACE treatment in HCC patients with MVI following surgery. (3) Most of the research objects included in this study were from China, and the efficacy and global applicability of this finding to clinical treatment are still under discussion.

In conclusion, postoperative sequential TACE treatment can improve 1-year, 2-year, 3-year, and 5-year RFS and OS rates for patients with pathologically confirmed MVI following radical HCC resection. This study guides clinicians in selecting appropriate cases for adjuvant TACE treatment during the clinical diagnosis and treatment to maximize patient benefits.

### Supplementary Information

**Additional file 1:** **Supplemental Table 1.** Scoring on detailed NOS evaluation item for cohort study.

## Data Availability

The datasets used and/or analyzed during the current study are available from the corresponding author on reasonable request.

## Abbreviations

TACE: Transcatheter arterial chemoembolization
HCC: Hepatocellular carcinoma
MVI: Microvascular invasion
HR: Hepatectomy resection
RR: Relative risk
CI: Confidence interval
RFS: Recurrence-free survival
OS: Overall survival
NOS: Newcastle-Ottawa Scale
