# Supplementary material for: Efficacy of sequential TACE on primary hepatocellular carcinoma with microvascular invasion after radical resection: a systematic review and meta-analysis
Source: World J Surg Oncol. 2023 Sep 5;21:277. doi: 10.1186/s12957-023-03160-0 (PMC10478229; doi:10.1186/s12957-023-03160-0)
Supplement: Supplementary file 1 — Additional file 1: Supplemental Table 1. Scoring on detailed NOS evaluation item for cohort study. [file 12957_2023_3160_MOESM1_ESM.docx]

**Supplemental table 1. Scoring on detailed NOS evaluation item for cohort study.**

| **First author, year** | **Selection** | | | | **Comparability** | | **Outcome** | | | **Total** |
| --- | --- | --- | --- | --- | --- | --- | --- | --- | --- | --- |
|  | **Representativeness of the exposed cohort** | **Selection of the non-exposed cohort** | **Ascertainment of exposure** | **No outcome of interest present at start of study** | **Comparability of cohorts on the basis of the design or analysis** | **Comparability of cohorts on the basis of the measurement** | **Assessment of outcome** | **Adequacy of follow up of cohorts** | **Completeness of follow up** |  |
| Wei W, 2019 [17] | 0 | 1 | 1 | 1 | 0 | 1 | 1 | 1 | 0 | 6 |
| Tian BY, 2018 [18] | 0 | 1 | 1 | 1 | 1 | 1 | 1 | 1 | 1 | 8 |
| Liu JH, 2016 [19] | 0 | 1 | 1 | 1 | 1 | 1 | 1 | 1 | 0 | 7 |
| Liu ZY, 2017 [20] | 0 | 1 | 1 | 1 | 0 | 1 | 1 | 1 | 0 | 6 |
| Shen PC, 2020 [21] | 0 | 1 | 1 | 1 | 1 | 1 | 1 | 1 | 0 | 7 |
| Qi YP, 2019 [22] | 0 | 1 | 1 | 1 | 1 | 1 | 1 | 1 | 0 | 7 |
| Li KW, 2012 [23] | 0 | 1 | 1 | 1 | 1 | 1 | 1 | 1 | 0 | 7 |
| Ye JZ, 2017 [24] | 1 | 1 | 1 | 1 | 1 | 1 | 1 | 1 | 1 | 9 |
| Wang L, 2020 [25] | 0 | 1 | 1 | 1 | 1 | 1 | 1 | 1 | 1 | 8 |
| Wang H, 2018 [26] | 1 | 1 | 1 | 1 | 1 | 1 | 1 | 1 | 1 | 9 |
| Sun JJ, 2016 [27] | 1 | 1 | 1 | 1 | 1 | 1 | 1 | 1 | 1 | 9 |
| Wang YY, 2019 [28] | 0 | 1 | 1 | 1 | 1 | 1 | 1 | 1 | 0 | 7 |
| Liu S, 2019 [29] | 0 | 1 | 1 | 1 | 1 | 1 | 1 | 1 | 1 | 8 |
